# Forensic mental health services: Current service provision and planning for a prison mental health service in the Eastern Cape

**DOI:** 10.4102/sajpsychiatry.v22i1.787

**Published:** 2016-05-06

**Authors:** Kiran Sukeri, Orlando A. Betancourt, Robin Emsley, Mohammed Nagdee, Helmut Erlacher

**Affiliations:** 1 Department of Psychiatry, Walter Sisulu University, South Africa; 2 Department of Psychiatry, University of Stellenbosch, South Africa; 3 Department of Psychology, Rhodes University, South Africa

## Abstract

**Objectives:**

No research data exists on forensic psychiatric service provision in the Eastern Cape, Republic of South Africa. The objective of this research was to assess current forensic psychiatric service provision and utilisation rates at Fort England Hospital. This is important in improving and strengthening the service. A related objective was to develop a model for a provincial prison mental health service.

**Methodology:**

This study is a situational analysis of an existing forensic psychiatric service in the Eastern Cape. The design of the study was cross sectional. An audit questionnaire was utilised to collate quantitative data, which was submitted to Fort England Hospital, Grahamstown. A proposed prison mental health service was developed utilising prevalence rates of mental illness among prisoners to calculate bed and staff requirements for an ambulatory and in-patient service.

**Results:**

During the study period a total of 403 remand detainees were admitted to the forensic psychiatry division of Fort England Hospital. The average length of stay was 494 days and the bed utilisation rate was determined at 203.54%. We estimate that to provide a provincial prison mental health service to treat psychotic illnesses and major depression the province requires a 52 bedded facility and a total staff complement of approximately 31.

**Conclusions:**

Forensic psychiatric services include the assessment, management and treatment of mentally disordered persons in conflict with the law and prisoners requiring psychiatric assessments. The Eastern Cape Province does not have plans or policies to assess and manage mentally ill offenders, resulting in an increased load on available services. We recommend that an inter-departmental task team, which includes Health, Justice and Constitutional Development and Correctional Services, should be established in the province, to develop a strategy to assist in the development of an effective and efficient forensic psychiatric service. This should be driven by the provincial Department of Health.

## Introduction

The Royal College of Psychiatrists^1^ defines forensic psychiatry as a specialty within psychiatry concerned with helping people who have a mental disorder and who present with a significant risk to the public. It covers areas such as, the assessment and treatment of mentally disordered offenders and prisoners. In South Africa, the Colleges of Medicine has recently recognised Forensic Psychiatry as a subspecialty.^2^

Forensic psychiatric services exist at the interface between mental health and the criminal justice system sectors. In terms of Chapter 13 of the *Criminal Procedure Act 51 of 1977* section 78(2)^3^, the justice sector identifies those who have mental health problems and they are assessed in a mental health setting and formal reports are provided. Chapters 6 and 7 of the *Mental Health Care Act*, no 17 of 2002^4^ make provision for the management of State patients (people who have committed an offence and who have been found to be unfit to stand trial or not criminally responsible as a result of mental illness) and mentally ill prisoners respectively. In addition to the above, the Department of Correctional Services identifies prisoners who may require mental health assessment and treatment and in terms of the *Correctional Services Act*^5^, it is the responsibility of the Department of Health to provide health services to prisoners.

In the Eastern Cape forensic psychiatric services are provided by Komani Hospital (single psychiatrist observations only) and Fort England Hospital (FEH), Grahamstown.^6^ The latter hospital conducts the majority of psycho-legal evaluations in the province. A task team set up by the National Department of Health found that there was no dedicated observation unit at Komani Hospital and that accommodation was shared by ‘observanda’ (defined as 30 day psychiatric evaluation of awaiting-trial prisoners, as per sections 77, 78 and 79(2) of the *Criminal Procedure Act*^3^) and State patients. The task team’s macro recommendation was that Komani Hospital required a new unit.^7^ The nature of services provided at FEH^6^ are as follows:

- The psycho-legal evaluation of observanda referred by courts in terms of sections 77, 78 and 79 of the *Criminal Procedure Act.*^3^
- The accommodation of State Patients in terms of section 42 of the *Mental Health Care Act*.^4^
- Admission and management of mentally ill prisoners.
- This comprehensive forensic psychiatric service provided only by FEH is for a population of 6 743 800.^8^ The hospital is located in the Cacadu District Municipality.

The forensic section of FEH consists of 189 designated male beds: 35 in each of four State patient wards and 49 beds in the maximum security unit (20 ‘observanda’ and 29 State patient or mentally ill prisoners). Female observanda and State patients are admitted to the generic female ward. New observanda and State patients are admitted either to the maximum security unit, Ward H (male State patients admission ward) or the generic female ward.

Mentally ill prisoners requiring ambulatory care are referred to the nearest mental health unit to the prison in which they are held.

The Eastern Cape has 45 correctional centres. At the time of the study (January 2010 to December 2010) the prison population in the Eastern Cape stood at 19 265 (449 females, 18 816 males). The national prison population for the same period was 160 545. The province has the second highest occupancy rate in the country (146.35%, the country average being 135.87%).^9^ The province does not have a prison mental health service, prison liaison or in-patient services and there are no services for children and adolescents in the youth justice system. Only two correctional centres report that a psychologist is consulted as needed. No correctional centre has a resident or visiting psychiatrist.^9^

The province does not have a long term care policy for the management of Sate patients and/or mentally ill prisoners i.e. for the integration of these patients into non-forensic services (hospital and community based). None of the other five institutions^10^ have beds set aside for the admission of mentally ill prisoners and/or State Patients. No supportive accommodation exists for mentally ill prisoners on parole and there are no community forensic services in the province to assist with the care and rehabilitation of State Patients.

It is well established among several population groups that the prevalence of severe mental illness differs between the general population and the prison population, with higher rates in the prison population.^11,12,13^ In a study conducted by Fazel and Danesh^14^, 1 in 7 prisoners in Western countries had a psychotic illness or major depression. It has also been demonstrated that deliberate self harm and completed suicide rates are higher among the prisoners than the general population.^15,16,17^

Singleton et al.^18,19,20^ have conducted detailed analysis of prevalence rates of psychiatric disorders in prison populations in England and Wales. A systematic review with severe exclusion criteria was done by Fazel and Danesh.^14^ This study included a varied population from countries such as Spain, United Kingdom, Australia, New Zealand and Canada. It included a varied sample of standardised interview tools such as the Structured Clinical Interview for the Diagnostic and Statistical Manual, Schedule for Assessment in Neuropsychiatry and the Structured Clinical Interview for Diagnostic and Statistical Manual Personality Disorders. The study covered a prison population of 22 790 and the findings were that 30.55% of men had anti-social personality disorder, 10% had depression and 3.7% had a psychotic illness. Among women prisoners the study found that 9% had anti-social personality disorder, 4% had depression and 12% had a psychotic illness.

In utilising the General Health Questionnaire (GHQ) and the Depression Subscale of the Hospital Anxiety and Depression Scale, Fatoye et al.^13^ found that 87.8% of their prison population sample had possible psychiatric morbidity on the GHQ and 85% of inmates had clinically significant depressive symptoms. In another Nigerian study^21^, conducted seven years later and utilising the GHQ and Composite International Diagnostic Interview (CIDI), a 57% prevalence rate of psychiatric disorders was discovered, with substance use disorder and depression rated as 48.75 and 30.8% respectively. A Zambian^22^ study using the Self Reported Questionaire-20 (SRQ-20) found a 63.1% prevalence rate of psychiatric illness. In South Africa, Naidoo and Mkize^23^ conducted a study at the Westville Correctional Centre in Durban. These investigators, using the Mini International Neuropsychiatric Interview (MINI), found a 55.4% prevalence rate of Axis I disorders, with substance use disorders at 23.3% and a life time prevalence of depression at 24.9%.

All four studies^13,21,22,23^ included awaiting-trial and sentenced prisoners. The sample sizes varied from 183 in the South African study to 206 and 186 in the Nigerian and Zambian studies respectively.

The objective of this research was to assess current forensic psychiatric service provision and utilisation rates at Fort England Hospital. This is important in improving and strengthening the service to provide a comprehensive service that is geographically equitable in the province. A related objective was to develop a model for a provincial prison mental health service.

## Methodology

The study was a situational analysis of forensic psychiatric service provision at FEH, Eastern Cape. The study used cross sectional design. The study period was from January 2010 to December 2010.

The study was conducted in two inter-related parts:

1. A questionnaire designed to collect data on the number of observations conducted, State patients admitted, average length of stay and staff categories and numbers employed during the study period was submitted to the forensic psychiatry service at FEH for comment and edition. The questionnaire was completed by a senior psychiatrist working in the Forensic Psychiatry section at FEH.
  The bed utilisation rate at FEH was calculated as follows: number of observanda admitted/capacity*100.
2. In calculating psychiatric service needs for a provincial prison mental health service (PMHS) the entire prison population was utilised i.e. awaiting trial and sentenced prisoners. Kaliski reported at the Infrastructure Unit Support System (IUSS) Mental Health Workshop that no national and international norms and standards for forensic psychiatry exist because patient populations differ.^24^ The Norms Manual for Severe Psychiatric Conditions^25^ was utilised to calculate service requirements for a prison population. Pages 26–39 of this manual delineate the stepwise calculations for service needs. In calculating staff requirements, Full Time Equivalents (FTE) were calculated, i.e. the number of staff that work full time in the forensic unit, which includes percentages of those staff who spend only some of their time in the forensic unit. In the development of service needs requirements for the prison population, the following was calculated:
  1. Annual ambulatory care visits (out-patient visits) at 100% coverage: Annual visits = prevalence × target population × coverage × minimum annual visits/person.^25^
  2. Utilisation rate/person/year = total ambulatory care visits/total population.^25^
  3. Daily patient visits [the number of patients who make use of an outpatient service/day] = total annual visits/working days per year.^25^
  4. Number of beds needed = number of severe cases × % needing hospitalization × (ALOS/365) × rotation factor.^25^
  5. Human resource needs were calculated for ambulatory care, acute and medium long stay facilities and managerial requirements.^25^

In order to determine where forensic and prison mental health services should be situated, the number of correctional centres and their approved capacity, the number of magistrate and high courts was determined. This was then grouped according to the five main locations where mental health facilities are available in the province, viz. Queenstown, Mthata, East London, Grahamstown and Port Elizabeth.

## Results

The results are presented in two parts. Part 1 demonstrates the results of the situational analysis of services at FEH and Part 2 the service needs requirements for a provincial Prison Mental Health Service (PMHS).

### Part 1: Service Provision at FEH: January 2010-December 2010

A total of 403 observanda were admitted [394 male, 9 female], 12 of whom were adolescents. Adjusted to population figures, this is a rate of 5.97 per 100 000. The average length of stay was 494 days.

The bed utilisation rate was calculated at 203.54%.

It is difficult to calculate bed/population ratios as the number of observanda are unpredictable. If this calculation is based on the number of admissions then the ratio would be 189/394 [0.48] for males. The ratio for females cannot be calculated as they are admitted to the generic female ward.

The staff distribution is shown in Table 1. It is important to note that nurses provide general nursing skills only. There are no nurses with advanced training in forensic psychiatry. The nurse/patient ratio was 1:4.

**TABLE 1:** FTE staff of the Forensic Section of FEH.

Staff type	FTE in forensic unit
Nurse	112
Clinical Psychologist	6
Medical Officer	1.5*
Registrar	0.9**
Psychiatrist	1.6***
Social Worker	1
Occupational Therapist	1
OTA*#	0

**Total**	**124**

# OTA, Occupational therapist assistant;

* 5 medical officers at 0.3 FTE;

** 3 registrars at 0.3 FTE;

*** 1 psychiatrist full time and 2 at 0.3 FTE.

### Part 2: Provincial Prison Mental Health Service

A prevalence rate of 3.7% and 4.0% for psychotic illness in males and females respectively and 10% and 12% for major depression was utilised.^14^ The results are demonstrated in Table 2.

**TABLE 2:** Expected prevalence of mental illness in the prison population in the Eastern Cape.

Mental Illness	Males [*N* = 18816]	Females [*N* = 449]	Total
Psychotic illness	693	18	**714**
Major Depression	1882	54	**1936**

**Total**	**2578**	**72**	**2650**

The results from Table 2 indicate that the target population for the development of a model for prison mental health services was 2650.

Service needs requirements were calculated as set out in the methodology.

1. Annual ambulatory care visits/outpatient visits: Males = 4 238.23, Females = 138.24 giving a total of 4376.5.
2. Utilisation rate/person/year = 0.23 at 100% coverage.
3. Daily patient visits to outpatient clinics = 16.58.
4. Number of beds needed: Total of 52 beds [consisting of 32 male and 1 female for acute stay and 19 beds for medium-long term stay].
5. Human resource needs are demonstrated in Tables 3–7. Staff requirements were calculated for an ambulatory, acute stay, medium long stay service, as well as for managerial requirements.

**TABLE 3:** Staff required for an ambulatory PMHS.

Staff type	Number required
Psychiatric nurse	0.67
General nurse	1.67
Occupational therapist	0.20
Ota	0.50
Social worker	0.30
Clinical psychologist	0.30
Psychiatrist	0.25
Registrar/medical officer	0.25

**TABLE 4:** Staff required for a 33 bedded acute PMHS.

Staff type	Number required
Psychiatrist [head of unit]	1.2
Registrar/medical officer	1.2
Social worker	0.60
Psychologist	0.60
Nurses	14.00

**TABLE 5:** Staff required for a 19 bedded medium long stay PMHS.

Staff type	Number required
Psychiatrist[head of unit]	0.20
Registrar/medical officer	0.40
Psychologist	0.40
Social worker	0.40
Occupational therapist	0.20
Ota	0.80
Nurses	6.10

**TABLE 6:** Managerial requirements for a PMHS.

Staff type	Number required
Chief regional mental health professional	0.50
Nurse manager	0.03

**TABLE 7:** Total staff requirements for a comprehensive PMHS.

Staff type	Inpatient	Ambulatory	Managerial	Total
Nurses	20.10	2.34	0.03	**22.47**
OT	0.40	-	-	**0.40**
Ota	1.30	-	-	**1.30**
Social workers	1.00	0.30	-	**1.30**
Psychologists	1.00	0.40		**1.40**
Psychiatrists	1.40	0.25	0.50	**2.15**
Registrar/MO	1.60	0.25	-	**1.85**

**Total**	**26.80**	**3.37**	**0.53**	**30.70**

**TABLE 8:** Justice and Correctional facility distribution in the Province.

Major mental health facility	Number of magistrate courts	Number of high courts	Number of correctional centres	Approved prison population
Mthata	15	1	19	2206
East London	7	1	7	3006
Grahamstown	3	1	2	471
Queenstown	5	0	9	2427
Port Elizabeth	9	1	8	5082

**Total**	**39**	**4**	**45**	**13192**

Total human resource needs are reflected in Table 7.

## Discussion

There is a dearth of published research in forensic psychiatric service planning and evaluation in South Africa. The Eastern Cape Department of Health does not have a forensic psychiatry strategy. Although FEH has the fourth largest forensic psychiatry section in the country^26^, the results of this study indicate an overburdened service. Strategies need to be devised to improve the current use of this service.

Mkize et al.^27^ recommended that a distinction be made between minor and major crime. The authors suggested that those who have committed a minor offence should be assessed by a district surgeon prior to referral to psychiatric services and those who have committed a major offence should be referred to Fort Napier Hospital. The authors further recommended that to decrease the number of State patients in Kwa-Zulu Natal, the Department of Health in that province develops twenty bedded forensic units in specific regions in that province. A total of 290 forensic beds were recommended. Mkize et al.^27^ also recommended that all ‘dangerous mentally ill’ patients in Kwa-Zulu Natal be referred to Fort England Hospital. This recommendation was not followed through with. This practice would have increased the service utilisation rate at Fort England Hospital and severely impacted forensic psychiatric service delivery for the Eastern Cape.

In 2010, a National Department of Health^8^ report found that ‘it was legally compatible to conduct single psychiatrist forensic observations in prisons or on an out-patient basis in health establishments.’ The report concluded that observation cases need not be admitted to a psychiatric hospital for 30 days and that reports generated by this practice were acceptable in Courts. As of March 2010, single psychiatrist observations were conducted in the Northern Cape (in detention centres), North West Province, Limpopo and Free State.^7^ A study conducted in Limpopo Province showed that 85% of observandi could be assessed on an outpatient basis by a multi-disciplinary team.^28^ This would reduce waiting lists for patients referred for observation. The same study reported that this system had decreased the number of patients in police cells and improved collaboration with police and justice departments. The National Department of Health Report^8^ supported this practice of outpatient observations and instituted a pilot project in February 2010 at Sterkfontein and Weskoppies Hospital. In April 2010 the number of single psychiatrist observations at Weskoppies Hospital dropped from forty to zero and the waiting period was reduced to seven days.^7^

Whilst there are several merits to these models, there are also a number of potential drawbacks. The quality and reliability of out-patient assessments depend on several factors; such as the availability of a full multi-disciplinary team and at least two psychiatrists (in cases involving serious charges), reliable collateral information, first language interpreters, psychometric testing and functional assessments on site. Out-patient assessments lack the benefit of longitudinal nursing observations. Consideration could however be given to limiting such cross-sectional outpatient assessments to offences not involving serious violence and permitting single psychiatrist reports in such cases.

An additional factor to assist in reduction of utilisation rates would be to increase bed capacity and the accompanying staff numbers or the development of community forensic facilities in areas where patients reside. The major drawback of community centres in this province is the distances from the main centres and the lack of human resources. An alternative would be to increase the number of beds at the five identified major centres and designate these for State patients. An increase of 200 beds for State patients will significantly decrease the utilisation rate at FEH and allow for easier reintegration of patients into their communities. The location of the centres in five urban areas in the Eastern Cape is in keeping with Kaliski’s recommendations, as they would attract qualified personnel, are close to tertiary hospitals and have easy access for patients and the public.^23^

The Ontario Ministry of Health and Long Term Care^29^ recommended 5.2 beds/100000 adults for designated regional secure beds (in a South African context these refer to State patient beds). The Eastern Cape currently has 2.96 beds per 100000 and the proposed additional beds will increase this figure to 5.93 beds per 100000, which will put the province on par with developed nations in the provision of State patient beds.

A model needs to be developed that will decrease State patient load at FEH and allow for relocation of patients to their districts of origin for assessment and further care and rehabilitation. A possible approach would be the utilisation of an integrated model. In this model, medium and low secure beds would be attached to mental health units throughout the province. These units would provide in-patient care and once the need for security has ended, local services would take over management.^30^ These units would have to provide services for medium and low security patients.

Medium security is defined as the level of security necessary for patients who have been dealt with in courts and represent a serious but less immediate danger to others. These patients present with a risk of absconding.^31,32^ Low security is defined as the level of security deemed necessary for patients who present with a less serious danger to others and have already been through the court procedures.^31,32^ Security measures are intended to impede rather than to completely prevent patients from absconding. Medium and low secure beds have been developed in the United Kingdom. The Royal College of Psychiatrists has developed standards for medium secure units that could be adapted for this province.^33^ In the South African context these security measures would pertain to the development of locked decentralized State patient wards.

These measures would initially incur costs in building and human resource training, however in the long term they will result in cost effective measures to improve forensic psychiatric service use in the province. The National Tertiary Services Grant (NTSG)^34^ which provides a platform for the funding of a wide array of specialised medical services including the funding of psychiatric services, should be explored by the provincial department of health as a potential source to finance forensic psychiatric care. The funding of psychiatric services includes child and adolescent psychiatry and 22 other psychiatric disorders, which includes maximum security units and services for State Patients. At present The National Maximum Secure Unit at FEH is the only one funded by this grant in South Africa.^6^ There are four basic criteria required for qualification for the grant, which are that the unit be headed by a specialist, services provided by a specialist, be a designated operationally distinct unit within a hospital (including out-patients clinics) and be fully operational at the time of assessment for funding.^34^ In order to expand forensic services in this resource limited province and reduce geographical inequity, the provincial department of health should investigate the possibility of developing tertiary forensic psychiatric services at other sites in the province that could meet these criteria. Currently all mental health units in the province have specialists, however there are no designated beds for State patients. Therefore this strategy will require the construction of designated units for State patients only, as this is a major pitfall to service provision currently.

Collaboration between the public and private sectors could assist in reducing waiting periods for observations within the province, even though this is not a challenge at present. In the period 2009/2010 there were 25 psychiatrists in the private sector in South Africa that were conducting assessments/evaluations, of these five were in the Eastern Cape.^7^ One of the reasons cited for the low number of observations done in the private sector was the low tariffs paid by the Department of Justice and Constitutional Development. The National Department of Health had recommended that the tariff be revised in line with the National Reference Planning List.^7^

Another option to decrease forensic psychiatric service use at FEH and the incarceration of mentally ill persons would be the development of pre-trial services and mental health courts. The formation of a review board, working as an independent tribunal, as is done under Canada’s Criminal Code, may be a more cost saving mechanism than mental health courts. The Review Board’s mandate is to protect public safety while also safeguarding the rights and freedoms of mentally disordered persons accused of committing crimes.^35,36^ This review board is composed of a psychiatrist, lawyer and a mental health professional, a senior lawyer or retired judge. When an individual is found unfit to stand trial, the review board will then hold a hearing, within 45–90 days, to further assess the accused’s fitness to stand trial. If the individual is found fit to stand trial, they are then returned to the court and the case proceeds. If the individual is determined to be unfit to stand trial, the review board will make an order that the person be held in custody or discharged back into the community with certain restrictions on their freedom. Hearings are done in the community in which the accused lives and are open to the public.

This model of service provision could be a joint project between the Departments of Justice and Constitutional Development and Health. It could be set up in all regions of the province. The model is similar to current provisions of the *Mental Health Care Act*^4^ with regards to the establishment of review boards. The functions of current review boards could be expanded to align to this model. Both the Mental Health Care^4^ and *Criminal Procedure Act*^3^ will require amendments to implement this model. Its application will revolutionize the procedures involved in assessing the mentally ill offender and in the long term will decreases costs and improve patient care and access to care.

A prison telepsychiatry service is another method that could assist in decreasing the current forensic psychiatric load. Telepsychiatry is defined as the provision of mental health services across distances through the use of live sound and video images.^37^ This form of interviewing has been found to be acceptable by both general and forensic patients and the service can be easily set up in courts and correctional centres.^38,39,40,41^ Limitations of this service, in addition to issues of privacy and confidentiality, include the high maintenance costs of the technology used.

It is evident, from this research study that the provision of prison mental health services is complex, as it requires a detailed understanding of the prison system. This includes factors that impact on the mental health of prisoners, viz. prison conditions, sexual behaviour and gang formation. Currently in this province, prison mental health services are delivered by both the departments of correctional services and health. Health services in correctional centres are mainly delivered by nurses. This dual system results in a lack of access to records and communication between treating professionals is non-existent.

In this province, correctional centres are widely distributed; therefore a centralized prison mental health service would not be feasible. Authorities should consider the development of two centres, based in Mthata and Port Elizabeth, the regions with the largest prisoner populations respectively.

In the United Kingdom prison mental health is the sole responsibility of the Department of Health, however in a resource limited setting as the Eastern Cape an inter-departmental program (between the Departments of Justice and Constitutional Development, Health and Correctional Services) will decrease resource needs. To facilitate the increase in human resource needs these centres could function as training centres for psychiatrists, psychologists and other allied personnel. The appointment of psychiatrists and other staff should follow the appointment principles set out by the Department of Health in accordance with the relevant labour regulations. The Royal College of Psychiatrists^42^ recommends that the consultant psychiatrist should not become isolated within a full time prison post. Sessional posts are therefore preferable and in the model proposed the province will require four psychiatrists at 0.5 FTE.

Performance indicators for this service can be adopted from various countries, such as those set out by the National Health Service in the United Kingdom.^43^ These indicators would appraise the health environment, medicines management and discharge planning of offenders, which is important in improving integration of offenders into communities. Health care in these facilities should be provided as in community centres, with primary care physicians and nurses doing the initial screening and offenders requiring mental health services referred to specialised centres. A working committee should be identified to determine the appropriate services to be provided. The recommendations outlined in this article could serve as a working document for that committee. In the interim, measures need to be implemented to reduce the number of mentally ill offenders in correctional centres.

Police services are generally the first point of contact within the justice system. In US cities with a population of more than 100000 it was demonstrated that 7% of all contacts involved a person with a mental illness.^44^ It is therefore imperative to provide police offices with adequate training in the identification of mental illness and in the management of the mentally ill prior to transportation to a mental health facility. Thom has developed a detailed training module for this purpose.^45^ The training guidelines have been specifically developed to assist police officers in ‘handling situations where they are called to assist or deal with a person with a mental disorder.’ The guideline is divided into five sections and provides training scenarios and information on mental disorders.

Currently, the majority of correctional centres in the Eastern Cape do not have permanent psychological services and psychiatric services are non-existent. There are twenty two psychologists in all correctional centres in South Africa.^46^ None of the correctional centres have an onsite psychiatric unit. A serious implication of this is that an increasing number of mentally ill persons are incarcerated due to the lack of initial assessments.

This situation can be improved by the use of an appropriate screening method on prison entry. A routine screening for mental illness is critical for providing services and improving safety within correctional centres. Prisoners at risk for suicide can be identified with appropriate screening and separated from the general prison population. This screening can be conducted by nurses and primary health care physicians working in correctional centres. The Brief Jail Mental Health Screen [BJMS], is an eight-item scale, designed to determine the need for further mental health assessment. The BJMS has eight yes/no questions, takes two to three minutes to complete and is easy to administer.^47^ The instrument has a high accuracy among male offenders [which form the majority of the prison population in the province], of 74%. Its accuracy among female prisoners was shown to be 62%.^44,48,49^ This will allow for the ease of referral of mentally ill prisoners to appropriate mental health facilities. Training on the use of the BJMS can be provided by psychiatrists closest to correctional centres or this function can be outsourced by the Department of Correctional Services.

It is recognised worldwide that prisoners are entitled to all fundamental human rights, which include the access to healthcare. ‘The State has a total and inescapable duty to care for inmates in a manner that does not violate or compromise their constitutional rights.’^50^ Section 5 subsection 6 of the *Correctional Services Act*^4^ is clear on the provision of health care for prisoners. It states that a prisoner must as soon as possible after admission to a correctional centre be examined by a medical doctor or nurse. This practice will prevent what Kaliski^51^ refers to as ‘re-institutionalisation by stealth’, whereby families of persons with severe mental illness who display aggressive and/or violent behaviour are encouraged to lay charges against them, resulting in an increased burden on forensic mental health services.

## Conclusion

The clearly evident lack of comprehensive (i.e. observation units, State patient units and units for mentally ill prisoners) and satellite forensic psychiatric services throughout the Eastern Cape increases utilisation rate on current services. Appropriate planning and policy development of a comprehensive prison mental health service is not possible due to the absence of a centralized data base on mentally ill prisoners. There are several other factors that increase the demand on current forensic psychiatric services; these include the lack of treatment options in correctional centres, the increased use of the mental health system by the Justice Department and the criminalization of the mentally ill.

The Eastern Cape government should urgently develop protocols for the risk management, rehabilitation and discharge of the mentally ill who commit criminal offences and are incarcerated, prisoners who become mentally ill and the proper discharge of State patients and for the provision of community forensic services.

To address the planning and development of a comprehensive forensic psychiatric service it is recommended that an inter-departmental task team should be set up consisting of stakeholders from the Departments of Health, Justice and Constitutional Development and Correctional Services.

### Limitations

The prevalence rates for mental illness in a South African prison population are limited to one study conducted in Durban, South Africa, therefore the study by Fazel and Danesh was utilised.^14^ Results may not be generalizable to the Eastern Cape. The estimate of 2650 for a prison mental health service is a very conservative estimate as other severe mental illnesses have not been included in the study. Also comorbidities were not considered. Appropriate planning and policy development of a comprehensive prison mental health service in the Eastern Cape is difficult as there is no centralised data base on mentally ill offenders. The cross sectional design of the study is a further limitation.

## References

[1] Royal College of Psychiatrists Faculty of Forensic Psychiatry. http://www.rcpsych.ac.uk/workinpsychiatry/Faculties/forensic.aspx (accessed January 2011)

[2] Colleges of Medicine, South Africa Subspecialty certificate in Forensic Psychiatry of the College of Psychiatrists of South Africa: Cert Forensic Psychiatry (SA). http://www.collegemedsa.ac.za/view_college.aspx?examid=129 (accessed August 2014)

[3] South African Government Criminal Procedure Act 17 of 2001. Pretoria: Government Printer, 2001.

[4] South African Government Mental Health Care Act 17 of 2002. Pretoria: Government Printer, 2002.

[5] South African Government Correctional Services Amendment Act 25 of 2008. Pretoria: Government Printer, 2008.

[6] Van Rensburg BJ (editor) The South African Society of Psychiatrists (SASOP) and SASOP State Employed Special Interest Group (SESIG) position statements on psychiatric care in the public sector. S Afr J Psych 2012;18(3):133–148.

[7] Department of Health, Republic of South Africa A turnaround strategy to reduce the long waiting list and waiting period for forensic psychiatric observations/evaluations of awaiting trial detainees referred by courts in terms of section 77, 78 and 79 of the Criminal Procedure Act no. 51 of 1977. 2010: 1–14.

[8] Statistics South Africa Midyear population estimates, 2010. www.statssa.gov.za/midyearpopulation2010. (accessed May 2010)

[9] South African Government Judicial Inspectorate for Correctional Services. Annual Report 2010/2011. Treatment of inmates and conditions in Correctional Centres. http://judicialinsp.dcs.gov.za/annualreports/judicial_inspectorate_annaul%20%20report_2010-2011.pdf (accessed February 2011).

[10] Sukeri K, Alonso-Betancourt O, Emsley R Staff and bed distribution in public sector mental health services in the Eastern Cape, South Africa. S Afr J Psychiatr 2014;20(4):160–165.

[11] Eytan A, Haller DM, Wolff H, Cerutti B, Sebo P, Bertrand D, Niveau G Psychiatric symptoms, psychological distress and somatic comorbidity among remand prisoners in Switzerland. Int J Law Psychiatry 2011;34(1):13–19 21126766 10.1016/j.ijlp.2010.11.003

[12] Ogloff RPJ, Davis RM, Rivers G, Ross S The identification of mental disorders in the criminal justice system, no. 333, 2007. Australian Institute of Criminology, Australian Government http://crg.aic.gov.au/reports/2006-ogloff.pdf

[13] Fatoye FO, Fatoye GK, Oyebanji AO, Ogunro AS Psychological characteristics as correlates of emotional burden in incarcerated offenders in Nigeria. East Afr Medical Journal 2006;83(10):545–552. 10.4314/eamj.v83i10.9467 17310680

[14] Fazel S, Danesh J Serious Mental Disorders in 23 000 prisoners: A Systematic Review. The Lancet 2002;359:545–550. 10.1016/S0140-6736(02)07740-1 11867106

[15] Baillargeon J, Penn JV, Thomas CR, Temple JR, Baillargeon G, Murray OJ Psychiatric disorders and suicide in the nation’s largest state prison system. J AM Acad Psychiatric Law 2009;37:188–193. 19535556

[16] Rivlin A, Hawton K, Marzano L, Fazel S Psychiatric disorders in male prisoners who made near lethal suicide attempts: A Case Control Study. Br J Psychiatry 2010;197:313–320. 20884955 10.1192/bjp.bp.110.077883

[17] Marzano L, Fazel S, Rivlin A, Hawton K Psychiatric disorders in women prisoners who have engaged in near lethal self harm: a case control study. Br J Psychiatry 2010;197:219–226. 20807968 10.1192/bjp.bp.109.075424

[18] Singleton H, Meltzer H, Gatward R Psychiatric comorbidity among prisoners in the United Kingdom and Wales: Summary Report. London: Office for National Statistics; 1997.

[19] Lader D, Singleton N, Meltzer H Psychiatric morbidity among young offenders in England and Wales. London: Office for National Statistics; 2000. 10.1080/0954026021000046074 12745323

[20] O’Brien M, Mortimer L, Singleton N, Meltzer H Psychiatric comorbidity among women prisoners in England and Wales Further analysis of data from the 1997 ONS survey of psychiatric morbidity among prisoners in England and Wales. London: Office for National Statistics; 2001.

[21] Armiya’u AY, Obembe A, Audu MD, Afolaranmi TO Prevalence of Psychiatric morbidity among inmates in Jos maximum security prison. Open Journal of Psychiatry 2013;3:12–17. 10.4236/ojpsych.2013.31003

[22] Nseluke MT, Siziya S Prevalence and socio-demographic correlates for mental illness among inmates in Lusaka Central Prison, Zambia. Medical Journal of Zambia 2011;38(2):3–7, 1998.

[23] Naidoo S, Mkize DL Prevalence of mental disorders in a prison population in Durban, South Africa. Afr J Psychiatry 2012;15:30–35. 10.4314/ajpsy.v15i1.4 22344760

[24] Kaliski S Forensic Mental Health Services. Presentation at Infrastructure Unit Support Systems (IUSS) Mental Health Workshop, 2011 http://www.iussonline.co.za/iuss/wp-content/uploads/2011/12/Sean-Kaliski-Forensic-Mental-Health-Services-30-Nov-20111.pdf [accessed February 2012]

[25] Norms Manual for Severe Psychiatric Condition. Tender no. GES 105, 1997-1998

[26] Gauteng Department of Health Service Transformation Plan 2010-2020. 3rd Draft, October 2010.

[27] Mkize DL, Green-Thompson RW, Ramdass P, Mhlaluka G, Dlamini N, Walker J Mental Health Services in Kwa-Zulu Natal. S Afr J Psych 2004;10(1):8–12.

[28] Ndala M An Analysis of offenders referred for forensic evaluation in Limpopo, January 2005 to December 2006. Thesis submitted for Masters in Medicine (Psychiatry), University of Limpopo, 2009.

[29] Arboleda-Florez J Forensic Mental Health Expert Advisory Panel (Ontario) Assessment, treatment and community reintegration of the mentally disordered: final report/ Forensic mental health expert advisory panel for the Ontario Ministry of Health and Long Term Care. Toronto: Forensic Mental Health Expert Advisory Panel: 2002 http://www.hsjcc.on.ca/Resource%20Library/Forensic%20System%20and%20Reports/Assessment,%20Treatment,%20and%20Community%20Reintegration%20of%20the%20Mentally%20Disordered%20Offender%20-%202002.pdf

[30] Jobbins C, Abbott B, Brammer L, Doyle MA, McCann G, McLean R Best Practice Guidance: Specification for adult medium-secure service Health Offender Partnerships, Department of Health United Kingdom, Crown Copyright 2007.

[31] Definitions of security levels in inpatient facilities in Scotland Forensic Network, National Health Service, Scotland, United Kingdom, 2004 http://www.forensicnetwork.scot.nhs.uk/documents/previous_reports/LevelsofSecurityReport.pdf

[32] SSNDS Definition no.22 Specialised Mental Health Services (all ages) Third edition, 2009 Department of Health, United Kingdom Available at http://www.yhscg.nhs.uk/SSNDS-Version-3/22%20Specialised%20Mental%20Health%20v3.pdf

[33] Standards for medium secure units Quality network for medium-secure units. Edited by Tucker Sarah and Hughes Tessa, 1^st^ edition, 2007 Royal college of Psychiatrists http://www.rcpsych.ac.uk/pdf/Final%20Standards%20for%20Medium%20Secure%20Units%20PDF.pdf

[34] Department of Health, Republic of South Africa National Tertiary Services Grant Definitions. Pretoria, Government Printer: 2011

[35] Forensic Psychiatric Services Information for families. British Columbia Schizophrenia Society and Forensic Psychiatric Service Commission. British Columbia Mental Health and Substance Use Services, 2004 http://www.bcmhsus.ca/includes/download.php?file=../content/318/BCSS_FPS_FamilyInformationBooklet15NOV11.pdf

[36] Bettridge S, Barbaree H The Forensic Mental Health System in Ontario: An Information Guide. Centre for Addiction and Mental Health, Canada 2008 http://www.camh.ca/en/education/about/camh_publications/Pages/forensic_menthealth_infoguide.aspx

[37] Jones RM, Leonard S, Birmingham L Setting up a telepsychiatry service. Psychiatric Bulletin 2006;30:464–467

[38] Brodey BB, Claypoole KH, Motto J, Arias RG, Goss R Satisfaction of forensic patients with remote telepsychiatric evaluation. Psychiatric Services 2000, 51(10):1305–1307. 11013332 10.1176/appi.ps.51.10.1305

[39] Wynchank S, Fortuin J Telepsychiatry in South Africa: present and future. S Afr J Psych 2010;16(1):16–19.

[40] Miller TW, Buton DC, Hill K, Luftman G, Veltkamp LJ, Swope M Telepsychiatry: Critical dimensions for forensic services. J Am Acad Psych Law 2005;33:536–546. 16394233

[41] Mars M, Ramlall S, Kaliski S Forensic Telepsychiatry: a possible solution for South Africa. Afr J Psychiatry 2012;15:244–247. 10.4314/ajpsy.v15i4.31 22829226

[42] Prison Psychiatry: Adult Prisons in England and Wales College Report CR141, February 2007. Royal College of Psychiatrists, London http://www.rcpsych.ac.uk/usefulresources/publications/collegereports/cr/cr141.aspx

[43] Prison Health: Quality and Performance Indicators, Guidance Notes National Offender Management Service, Department of Health, United Kingdom, 2009.

[44] Gattiker E Mental Health and Law Enforcement Encounters: A Review of the Current Problem and Recommendations. Georgia Association of Chiefs of Police, Mental Health Ad Hoc Committee to Address Mental Health Issues in Law Enforcement, United States, 2007 http://www.gachiefs.com/pdfs/NEWS_GACP%20Mental%20Health%20Report.pdf

[45] Thom R The Mental Health Care Act 2002: Training guidelines for the South African Police Services, Directorate of Mental Health and Substance Abuse, National Department of Health, Republic of South Africa, 2003

[46] NICRO submission: Department of correctional Services Budget Vote 21, 2011 http://www.issafrica.org/crimehub/uploads/110316nicro_0.pdf

[47] Ford J, Trestman RL, Osher F, Scott JE, Steadman HJ, Robbins PC Mental Health Screen for Corrections. US Department of Justice, Office of Justice Programs, United States 2007 Available at https://www.ncjrs.gov/pdffiles1/nij/216152.pdf

[48] Steadman HJ, Robbins PC Developing and evaluating a brief jail mental health screen for women, final report. Abstract available at http://www.ncjrs.gov/App/Publications/abstract.aspx?ID=241907

[49] Evans C, Brinded P, Simpson AL, Frampton C, Mulder RT Validation of brief screening tools for mental disorders among New Zealand prisoners. Psychiatric Services 2010;61(9):923–928 20810592 10.1176/ps.2010.61.9.923

[50] Muntingh L Prisons in a Democratic South Africa - A guide to the rights of inmates as described in the Correctional Services Act and Regulations. Civil Society Prison Reform Initiative, Community Law Centre, Cape Town 2006 (revised 2010).

[51] Kaliski S Re-institutionalisation by stealth: The Forensic Mental Service is the new chronic system. Afr J Psychiatry 2013;16:13–17 10.4314/ajpsy.v16i1.2 23417630

